# Advances in Gastroesophageal Reflux Disease Management: Exploring the Role of Potassium-Competitive Acid Blockers and Novel Therapies

**DOI:** 10.3390/ph18050699

**Published:** 2025-05-09

**Authors:** Katarzyna Hossa, Ewa Małecka-Wojciesko

**Affiliations:** Department of Digestive Tract Diseases, Medical University of Lodz, 90-419 Lodz, Poland; katarzyna.hossa@stud.umed.lodz.pl

**Keywords:** GERD, PPIs, PPI-refractory GERD, P-CABs, LES, esophageal motility, refractory GERD, long-term relief, quality of life, vonoprazan, tegoprazan, fexuprazan

## Abstract

Gastroesophageal reflux disease (GERD) is a prevalent chronic gastrointestinal disorder that affects a substantial proportion of the global population. It is characterized by the extensive backward flow of stomach contents into the esophagus, leading to troublesome symptoms and potential complications. Proton pump inhibitors (PPIs) have long been the cornerstone of pharmacological treatment for GERD, effectively suppressing gastric acid secretion. However, a substantial subset of patients, referred to as PPI-refractory GERD, experience inadequate symptom control despite optimal PPI therapy. GERD significantly impacts patients’ quality of life, affecting domains, such as vitality, pain, and physical functioning. Consequently, there is an urgent need for alternative therapeutic strategies and novel pharmacologic agents to provide more effective, long-term relief. Emerging treatment options include potassium-competitive acid blockers (PCABs) like vonoprazan, which offer more potent and sustained inhibition of gastric acid secretion compared to traditional PPIs. Additionally, prokinetic agents such as itopride have gained attention due to their potential to improve GERD symptoms by enhancing gastrointestinal motility and accelerating gastric emptying. This article reviews the mechanisms of action, clinical efficacy, and potential of these novel therapeutic approaches in improving patient outcomes in GERD management. With the growing prevalence of PPI resistance and side effects, a personalized, multifaceted approach to treatment is becoming increasingly necessary to optimize care for patients with GERD.

## 1. Introduction

Gastroesophageal reflux disease (GERD) is a prevalent chronic gastrointestinal disorder characterized by the extensive backflow of stomach contents into the esophagus, leading to troublesome symptoms and, in some cases, complications [1]. The hallmark symptoms of GERD include heartburn and regurgitation, which are typically exacerbated by the supine position, bending over, or after consuming large meals. In addition to these classic esophageal symptoms, GERD may present with extraesophageal or atypical symptoms, such as chronic cough, hoarseness, wheezing, and non-cardiac retrosternal pain, which is the most common cause of chest pain unrelated to cardiac conditions [2]. GERD is a global health concern, with an estimated 13% of the general population experiencing symptoms at least once a week, with the prevalence being notably higher in South Asia and Southeast Europe (>25%) compared to regions like Southeast Asia, Canada, and France (<10%). In the United States, approximately 20% of the population reports weekly symptoms [3], with 7% experiencing daily symptoms, significantly impacting their quality of life [4]. The burden of GERD on patients’ physical and social well-being can be comparable to or even higher than conditions such as hypertension, duodenal ulcer disease, and post-heart attack recovery, particularly in domains of pain and physical functioning [5]. GERD can present in several phenotypes, including non-erosive reflux disease (NERD), which accounts for 60–70% of the patients and is characterized by symptoms without visible mucosal damage; erosive esophagitis (EE), seen in approximately 30% of GERD patients, where mucosal damage is evident; and Barrett’s esophagus, affecting 6–12% of GERD patients, where intestinal metaplasia increases the risk of esophageal adenocarcinoma [6]. The mainstay of pharmacological treatment for GERD has traditionally been proton pump inhibitors (PPIs), which effectively suppress gastric acid secretion. However, up to 40–55% of the patients with GERD continue to experience persistent symptoms despite optimal PPI therapy, leading to the recognition of PPI-refractory GERD. Moreover, the frequent recurrence of symptoms after PPI discontinuation results in patient dissatisfaction and poor compliance [7]. H2 receptor antagonists (H2RAs) and antacids are commonly used for symptom relief, while prokinetic agents can be prescribed in patients with associated motility issues. These alternatives may be helpful in some cases, although they are generally less effective than PPIs in controlling acid reflux [8,9].

## 2. Traditional GERD Treatment: Proton Pump Inhibitors (PPIs)

### 2.1. Chemical Structure and Mechanism of Action

Proton pump inhibitors (PPIs) are prodrugs with an enteric coating, absorbed in the small intestine, and subsequently activated in the acidic environment of the parietal cells. Once activated, they irreversibly bind to the hydrogen–potassium ATPase (H^+^/K^+^-ATPase) pump in the membrane of parietal cells, effectively inhibiting gastric acid secretion [10]. While PPIs share a common mechanism of action, their chemical structures are not identical. These compounds feature heterocyclic structures with both a benzimidazole and a pyridine group, connected by a methylsulfinyl linkage. Additionally, various side chains enhance their pharmacokinetic and pharmacodynamic properties [11].

PPIs have a long half-life and a lower tendency to develop tachyphylaxis, in contrast to H2 receptor antagonists (H2RAs). PPI action typically lasts for about 24 h, providing effective acid suppression throughout the day. This makes PPIs the treatment of choice for GERD, especially in the management of erosive esophagitis and symptom control related to acid hypersecretion [10].

PPIs are classified into first- and second-generation agents. The first generation includes omeprazole, lansoprazole, and pantoprazole, whereas the second generation comprises esomeprazole (S-isomer of omeprazole), rabeprazole, dexlansoprazole, and ilaprazole (approved in South Korea and China). Second-generation PPIs are characterized by a faster onset of action, stronger acid suppression, and reduced dependence on CYP450 metabolism compared to earlier agents, resulting in improved therapeutic outcomes [12].

### 2.2. Overview of International Guidelines for GERD Management

According to the 2022 guidelines of the American College of Gastroenterology (ACG), the initial management of typical GERD symptoms consists of an empirical trial of a standard-dose proton pump inhibitor (PPI) once daily for eight weeks, taken 30–60 min before the first meal. If symptoms persist, optimization of the PPI therapy by verifying intake timing and possibly increasing to twice-daily dosing is recommended. Long-term maintenance with the lowest effective PPI dose is advised in erosive esophagitis. In patients without erosive disease or Barrett’s esophagus whose symptoms resolve, the discontinuation of PPIs or switching to on-demand therapy is suggested. Diagnostic endoscopy is recommended if alarm symptoms, atypical symptoms, or persistent symptoms occur, ideally after a 2–4-week discontinuation of the PPI therapy. In patients with nighttime symptoms, bedtime H2 receptor antagonists may be added, while prokinetics are reserved for those with gastroparesis. In patients with persistent symptoms despite optimized PPI therapy, the routine addition of adjunctive medical therapies is not recommended; instead, further diagnostic evaluation should be pursued to guide management [13].

In Europe, in the management of GERD, a standard-dose proton pump inhibitor (PPI) is recommended once daily for 4–8 weeks depending on disease severity. PPIs should be administered 30–60 min before the first meal of the day to maximize efficacy.

For patients with mild disease, such as non-erosive reflux disease (NERD) or Los Angeles grade A/B esophagitis, on-demand or intermittent PPI therapy may be considered after symptom control. For severe erosive esophagitis (Los Angeles grade C/D) or GERD with complications (e.g., Barrett’s esophagus), long-term continuous PPI maintenance therapy at the lowest effective dose is advised. In the case of incomplete response, therapeutic strategies include verifying the adherence and timing of PPI intake, increasing the PPI dose to twice daily, switching to a different PPI, or adding alginates or H2 receptor antagonists.

Diagnostic upper endoscopy is recommended in patients with alarm symptoms, while ambulatory 24 h pH or pH-impedance monitoring should be considered in those with atypical symptoms, refractory GERD, or candidates for anti-reflux surgery [4,14].

In Japan, initial treatment for severe erosive esophagitis (Los Angeles grade C/D) consists of vonoprazan 20 mg daily for four weeks, combined with lifestyle modifications and optional alginates or antacids for symptom relief. In responders, maintenance therapy includes vonoprazan 10–20 mg daily or combination therapy with prokinetics or Japanese herbal medicines; switching to a minimal PPI dose may be considered if good symptom control is achieved. In non-responders, vonoprazan 20 mg can be continued for up to eight weeks or combined with other treatments. For mild erosive esophagitis (LA grade A/B), initial treatment with a standard-dose PPI or vonoprazan 20 mg daily is recommended, with maintenance options including a minimal dose of PPI or P-CAB, on-demand therapy, or combination therapy. In PPI-resistant cases, escalation to a double-dose PPI or switching to vonoprazan is advised. For non-erosive reflux disease (NERD), a standard-dose PPI for four weeks is recommended initially, with maintenance on a minimal or on-demand dose of PPI or combination therapy. In refractory GERD, evaluation with impedance-pH monitoring and/or esophageal manometry is advised, and intensified medical therapy or surgery should be considered if inadequate acid suppression is confirmed [15].

### 2.3. Effectiveness of PPIs in GERD Treatment

PPI efficacy varies depending on the clinical presentation of the disease, with the highest response in erosive esophagitis (EE). In this group, 85.6% of the patients experience PPI with significant symptom relief as well as healing of the esophageal lining. PPIs are also effective in treating non-cardiac chest pain associated with GERD, with a response rate of 74.5%, and in cases of uncomplicated heartburn, where it yields around 70.3% [16].

However, the efficacy of PPIs is lower in patients with heartburn without esophagitis, with a response rate of 39.7%. In subjects with extraesophageal symptoms (such as chronic cough or laryngeal symptoms), the response rate is even lower, ranging from 14.7% to 18.1% [16].

PPIs are superior to H2RAs in managing GERD symptoms and healing esophageal mucosal damage. Meta-analyses have demonstrated that PPIs have a healing rate of 83.6% for esophagitis compared to 51.9% with H2RAs and 28.2% with placebo. Additionally, PPIs promote faster healing, with a rate of 11.7% per week versus 5.9% per week with H2RAs, and provide more rapid relief of heartburn (11.5% per week compared to 6.4% per week with H2RAs) [17].

### 2.4. Limitations of PPIs and Challenges in GERD Management

Despite their widespread use and efficacy, PPIs do not address all the aspects of GERD treatment. Up to 20–40% of the patients with advanced erosive esophagitis experience treatment failure, and approximately 40% of the patients with non-erosive reflux disease (NERD) do not respond to standard PPI therapy. Nighttime heartburn, which affects about 38% of GERD patients, also remains a challenge for PPI treatment. Moreover, in long-term maintenance therapy, relapse rates are high, with approximately 30% of the patients experiencing symptom recurrence [10,18].

Another challenge is the PPI-refractory GERD. Furthermore, PPI therapy may be less effective in individuals with CYP2C19 genetic polymorphisms, which alter drug metabolism. In such instances, alternative therapeutic approaches, such as higher-dose PPI regimens or adjunctive therapies, may be considered [13].

Long-term proton pump inhibitor (PPI) use has raised concerns regarding nutrient malabsorption, particularly of iron, calcium, and vitamin B12. However, clinical evidence remains inconclusive, with substantial variability across studies and populations [19]. Some studies report a 65% increased risk of vitamin B12 deficiency after two years of PPI use [20], whereas others, including long-term observational studies on esomeprazole and omeprazole, have demonstrated stable iron, calcium, and vitamin B12 levels over periods of up to 5 and 10–12 years, respectively [21]. Pantoprazole appears to have a more favorable impact on B12 metabolism compared to other PPIs [22].

Long-term PPI therapy has also been associated with a modest increase in the risk of fractures, chronic kidney disease (CKD), and infections such as community-acquired pneumonia and *Clostridioides difficile* [22,23,24,25,26]. The link between PPI use and cognitive decline remains uncertain, with some emerging evidence suggesting a potential association mediated by vitamin B12 deficiency [22,26]. However, only two studies have supported this connection, and the majority have not confirmed a consistent relationship [19,27,28].

Additionally, rebound acid hypersecretion and benign gastric mucosal changes (e.g., fundic gland polyps) have been observed following PPI withdrawal [29,30]. Drug interactions, particularly with warfarin, theophylline, and clopidogrel, have been reported more frequently with omeprazole, whereas pantoprazole is associated with a more favorable interaction profile [22,29].

Importantly, many of the adverse effects attributed to long-term PPI use are supported by low- or moderate-quality evidence and may be confounded by underlying comorbidities or concomitant medication use [19].

## 3. New Directions in the Pharmacotherapy of GERD

### 3.1. Potassium-Competitive Acid Blockers

The first potassium-competitive acid blocker (PCAB) introduced was revaprazan (YH1885, Revanex), which was approved in 2007 for use in India and South Korea. Revaprazan is primarily indicated for the treatment of gastritis and peptic ulcer disease, though it is not used in the treatment of GERD due to its limited ability to elevate gastric pH [31].

Following the introduction of revaprazan, several other PCABs have been developed and launched. Vonoprazan (TAK-438, Takecab), introduced in Japan in 2015, is the most notable of these [32]. Clinical studies on vonoprazan are more extensive compared to other P-CABs, making it a well-documented treatment option. Research suggests that vonoprazan offers a more potent and prolonged inhibition of gastric acid secretion compared to both PPIs and other P-CABs. This enhanced efficacy is believed to be due to two key factors: higher pKa, which allows for higher concentrations in the gastric glands compared to PPIs and other P-CABs, and slower clearance from these glands, along with a more gradual dissociation from the H^+^, K^+^-ATPase enzyme, which prolongs its action and improves its acid-reducing properties [33,34]. Vonoprazan has since been expanded to other Asian countries and is used to treat a range of conditions, including peptic ulcers, erosive esophagitis, and Helicobacter pylori eradication. Additionally, like PPIs, vonoprazan is indicated for the prevention of gastric mucosal injury in patients using NSAIDs and aspirin [32].

Vonoprazan became the first PCAB to receive approval from the U.S. Food and Drug Administration (FDA) in November 2023 for treating erosive GERD and associated heartburn. This was followed by another FDA approval in July 2024 for the treatment of heartburn in non-erosive GERD, marking the first major advancement in GERD therapy in over three decades in the United States [35].

In addition to vonoprazan, other PCABs have been introduced into the market. Tegoprazan (CJ-12420, K-CAB), launched in South Korea in 2019, has been approved for the treatment of erosive esophagitis and non-erosive reflux disease (NERD). Tegoprazan has also been introduced in Latin America as the second PCAB to be marketed outside of Asia [36,37].

Further developments in the PCAB class include fexuprazan (DPW14012) and keverprazan (H008), both of which were approved in South Korea and China, respectively. Fexuprazan received approval in 2021, while keverprazan was approved in 2023 [38,39].

From a chemical perspective, vonoprazan, fexuprazan, and keverprazan all share a sulfonylpyrrole structure, whereas tegoprazan is a benzimidazole derivative. Additionally, the salts used for these drugs vary: vonoprazan is formulated as a fumarate salt, while keverprazan is available as a hydrochloride salt [40].

In addition to the currently marketed PCABs, two compounds, linazapran glurate and zestaprazan, are in clinical development and show promise for future therapeutic options in the treatment of acid-related disorders [40].

PCABs have demonstrated the ability to provide more consistent and sustained acid suppression compared to PPIs, leading to improved clinical outcomes and enhanced quality of life for patients. This improvement in quality of life is primarily due to better control of symptoms such as heartburn, regurgitation, and dysphagia, as well as fewer gastric acid breakthrough episodes, particularly at night. Patients experience less discomfort, better sleep quality, and fewer restrictions on daily activities. In addition, because PCABs can be taken independently of meals, they further increase patient convenience and compliance, contributing to more effective management [40].

### 3.2. Pharmacodynamic Properties of Potassium-Competitive Acid Blockers (PCABs)

PCABs exert their therapeutic effects by reversible inhibition of the proton pump (H^+^/K^+^-ATPase), a key enzyme located in the parietal cells of the stomach, responsible for the final step in gastric acid production [7].

PCABs function by competitively binding to the potassium-binding site of the proton pump, preventing the exchange of potassium ions (K^+^) for protons (H^+^). This inhibition suppresses gastric acid secretion effectively. Unlike traditional proton pump inhibitors (PPIs), which are prodrugs requiring activation in an acidic environment, PCABs exert their effects directly upon absorption, offering a more rapid onset of action [7].

PCABs, due to their weak basic nature and lipophilic properties, remain stable in the acidic gastric environment. Their relatively high pKa values enable them to accumulate in the acidic secretory tubules of parietal cells, which enhances their ability to inhibit acid secretion [7].

Unlike PPIs, which can only inhibit the proton pump once it is activated by the presence of gastric acid, PCABs can inhibit both the activated and unactivated forms of the proton pump. This dual mechanism of action allows PCABs to provide a more consistent and sustained reduction in gastric acid secretion, independent of food intake or gastric acid stimulation. Consequently, PCABs offer superior acid control, both during the day and night, with no need for food intake stimulation [41].

In the management of GERD, maintaining a gastric pH of 4.0 or above is crucial for effective symptom relief and mucosal healing [42]. Randomized clinical studies conducted in Japan (*N* = 84) and the UK (*N* = 63) in healthy male volunteers aged 18 to 45 years demonstrated that vonoprazan significantly increases gastric pH in a dose-dependent manner compared to placebo. Continuous gastric pH monitoring was conducted over a 24 h period with intragastric probes. The participants received a single dose of vonoprazan, ranging from 1 to 120 mg in Japan and 1 to 40 mg in the UK. The results indicated that a 40 mg dose of vonoprazan maintained gastric pH ≥ 4 for 92% of the 24 h period in the Japanese cohort, as measured by the Holding Time Radio (HRT), while in the UK cohort, the same dose maintained pH ≥ 4 for 87% of the 24 h period. Notably, gastric pH remained ≥ 4 for 100% of the nocturnal period in the Japanese cohort and 90% in the UK cohort (both with a 40 mg dose), highlighting vonoprazan’s superior nocturnal acid suppression. In comparison, PPIs typically maintain a nocturnal pH ≥ 4 for up to 72% of the time, with this depending on dosage and duration [34].

In a randomized, multicenter study comparing the acid-suppressing effects of vonoprazan (20 mg once daily) with rabeprazole (20 mg and 40 mg daily, administered in two divided doses) in healthy adults, three cohorts (each consisting of 10 participants aged 18–45 years) underwent 7 days of therapy. Gastric acid suppression was assessed using 24 h pH monitoring with a multi-channel system, measuring pH levels in the stomach. The results demonstrated that vonoprazan 20 mg provided stronger and longer-lasting acid suppression compared to both doses of rabeprazole. In the first cohort (V20 vs. R20), vonoprazan maintained gastric pH ≥ 4 for 88.4% of the 24 h period, while rabeprazole maintained this pH for 53.8%. In the second cohort (V20 vs. R40), vonoprazan maintained pH ≥ 4 for 95.0% compared to rabeprazole at 74.5%. Nocturnal acid control was particularly pronounced with vonoprazan (20 mg), which maintained pH ≥ 4 for 91.5% of the night compared to 73.2% for rabeprazole (40 mg) [43].

In a randomized, open-label, crossover study, the acid-suppressing effects of vonoprazan 20 mg were compared with esomeprazole 20 mg and rabeprazole 10 mg over 7 days in 20 healthy men (all CYP2C19 metabolizers). Continuous pH monitoring was conducted using intragastric probes in the stomach over a 24 h period on Days 1 and 7. The results showed that vonoprazan provided significantly better acid suppression compared to both PPIs. On Day 1, the average 24 h pH ≥ 4 time was 71.4% for vonoprazan compared to 23.9% for esomeprazole. On Day 7, vonoprazan achieved 85.8%, while esomeprazole only reached 61.2%. In nighttime (nighttime suppression assessed during the 12–24 h period after dose administration), vonoprazan maintained pH ≥ 4 for 67.9% on Day 1 and 75.2% on Day 7 compared to 12.9% and 44.8% for esomeprazole. Similarly, vonoprazan showed better nighttime acid suppression compared to rabeprazole, with 84.3% on Day 1 and 88.8% on Day 7 compared to 15.3% and 54.1% for rabeprazole [44].

Tegoprazan, another PCAB, demonstrated significant potential in overcoming the limitations of traditional PPIs, particularly in terms of nighttime acid suppression. In a randomized, open-label crossover study, its acid-suppressing effects were compared with vonoprazan and esomeprazole. In this study, 16 healthy participants (6 CYP2C19 extensive metabolizers, 5 intermediate metabolizers, and 5 poor metabolizers) received single night doses of tegoprazan (50 mg), vonoprazan (20 mg), and esomeprazole (40 mg), orally around 10.00 PM, following at least 3 h of fasting. Continuous pH monitoring was performed using a gastric probe. Tegoprazan exhibited a rapid onset of action, increasing gastric pH > 4 within approximately 1 h after administration, while vonoprazan and esomeprazole effect was seen after up to 4 h. The percentage of nighttime hours with gastric pH ≥ 4 was 66.0% for tegoprazan, 60.5% for vonoprazan, and 36.1% for esomeprazole. Although tegoprazan showed a slightly higher percentage of nighttime hours with gastric pH ≥ 4 compared to vonoprazan, the difference between these two PCABs was minimal, suggesting comparable effectiveness in maintaining gastric pH suppression overnight. Both tegoprazan and vonoprazan showed no dependency on CYP2C19 polymorphisms, which is typical for esomeprazole [45].

The pharmacodynamics of tegoprazan were evaluated in a phase I randomized, double-blind, placebo-controlled clinical trial involving 56 healthy male participants. The study consisted of three parts: a single ascending dose (SAD) study, a multiple ascending dose (MAD) study, and a pharmacodynamic comparison with esomeprazole. In the SAD study, the participants (N = 32) received single oral doses of tegoprazan at 50, 100, 200, or 400 mg. In the MAD study, eight participants were given 100 or 200 mg of tegoprazan once daily for 7 consecutive days. In the pharmacodynamic comparison, eight participants received 40 mg of esomeprazole daily for 7 days. All the treatments were given under fasting conditions. Gastric acid suppression was measured by continuous 24 h intragastric pH monitoring using a nasogastric pH probe in each study [46].

The results showed that tegoprazan provided dose-dependent acid suppression, with significant increases in gastric pH observed approximately one hour after administration. In the SAD study, the percentage of time during which pH remained above four ranged from 48.9% at the 50 mg dose to 87.4% at the 400 mg dose, demonstrating a clear dose–response relationship. In the MAD study, prolonged acid suppression was observed with repeated administration, as the mean percentage of time with gastric pH > 4 was greater on Day 7 compared to Day 1. Specifically, for the 100 mg dose, pH > 4 increased from 62.3% on Day 1 to 70.4% on Day 7. For the 200 mg dose, pH > 4 increased from 76.8% on Day 1 to 94.6% on Day 7, indicating a stable and reliable suppression of gastric acid over time [46].

### 3.3. Pharmacokinetics of P-CABs: A Focus on Vonoprazan

Drugs in the P-CAB class, such as vonoprazan, have a significantly longer half-life (t_1_/_2_) compared to proton pump inhibitors (PPIs), ranging from 3.7 to 10.3 h, while PPIs typically have a half-life of 1 to 2 h [47]. Vonoprazan has a half-life (t_1_/_2_) of 7 to 9 h independent of food [48]. After oral administration, vonoprazan is rapidly absorbed, with the median time to reach maximum plasma concentration (C_max_) occurring within 1.5 to 2 h (t_max_) after a single dose regardless of the dose size [48,49]. The C_max_ of 37.8 ng/mL is reached after a single dose, and after a steady state is achieved with repeated dosing, the peak concentration (t_max_) is reached on average within 3 h [48].

Vonoprazan is primarily metabolized by the cytochrome P450 enzyme system, involving CYP2C9, CYP2C19, CYP3A4/5, CYP2B6, and CYP2D6. Its metabolites do not possess any pharmacological activity [48,49]

Pharmacokinetic studies of vonoprazan have not demonstrated any significant variability based on CYP2C19 metabolizer status. This suggests that vonoprazan’s metabolism is not influenced by genetic variations in the CYP2C19 enzyme, a notable advantage over other drugs like PPIs that may have variable efficacy depending on an individual’s metabolic profile [49].

Vonoprazan is mainly eliminated through the kidneys, with a significant portion (around 67%) excreted in urine, and a smaller portion (about 31%) in feces. Only a small fraction of the excreted drug is in its unchanged form, indicating extensive metabolism prior to elimination [48].

#### Influence of Food and Gastric Environment on Vonoprazan

Food intake has only an insignificant influence on vonoprazan pharmacokinetics. The high-fat meal results in a slight increase in the maximum plasma concentration by 5% and a delay in the time to reach maximum concentration to approximately 5 h compared to fasting conditions. Furthermore, vonoprazan’s half-life is unaffected by food intake [48]. Therefore, the impact of meal on vonoprazan’s therapeutic efficacy remains minimal. These findings confirm that vonoprazan can be administered regardless of food intake, ensuring stable therapeutic effects and patient convenience [48].

Vonoprazan’s stability and activity in the acidic gastric environment are critical to its mechanism of action. With a pKa of 9.06, vonoprazan remains stable and pharmacologically active even in the stomach’s acidic environment [48,50]. In the secretory canaliculi of the parietal cells (pH~1.0), the drug predominantly exists in its protonated form, which facilitates its accumulation in this location. Based on calculations, it is estimated that the concentration of protonated vonoprazan in the canaliculi can be up to 10^8^-fold higher than in plasma, where the pH is 7.4 and vonoprazan predominantly exists in its non-protonated form [49]. The drug’s high affinity for the H^+^, K^+^-ATPase (proton pump) enables it to effectively inhibit gastric acid secretion even at neutral pH, with an inhibition constant (Ki) of 10 nM at pH 7, indicating strong binding to the proton pump, which becomes even stronger at a slightly more acidic pH (Ki) of 3 nM at pH 6.5. Unlike traditional proton pump inhibitors (PPIs), vonoprazan remains effective over a wider pH range, providing consistent acid suppression regardless of fluctuations in stomach pH, ensuring stable therapeutic effects and reliable acid control [50].

The comparison of proton pump inhibitors (PPIs) and potassium-competitive acid blockers (P-CABs) is summarized in Table 1.

### 3.4. Efficacy of Vonoprazan in the Treatment of Reflux Esophagitis and GERD

#### 3.4.1. Efficacy in Reflux Esophagitis (RE) Healing

A prospective study by Hoshino et al. examined the efficacy of vonoprazan in treating PPI-resistant reflux esophagitis (RE). The study included 24 patients categorized with the Los Angeles (LA) classification, all of whom were unresponsive to at least 8 weeks of PPI therapy (lansoprazole 30 mg, rabeprazole 10 mg, or esomeprazole 20 mg daily in the morning). These patients, with endoscopically confirmed esophagitis, were treated with 20 mg of vonoprazan daily after breakfast for 4 weeks [51].

Follow-up endoscopic evaluations after 4 weeks showed that 87.5% of the patients achieved successful healing of the esophageal changes, with the success rates varying by disease severity: 100% for grade A (3/3), 85.7% for grade B (6/7), 90.9% for grade C (10/11), and 66.7% for grade D (2/3). Patients with additional pathologies, such as hiatal hernia or scleroderma, were less likely to achieve complete acid suppression. One patient with both scleroderma and a hiatal hernia (grade C RE) exhibited gastric pH levels below four for 52.2% of the treatment duration, which points out the challenges in acid control in complex cases. However, vonoprazan was highly effective in most patients, especially those without additional complicating factors [51].

Symptom relief, assessed using the Frequency Scale for Symptoms of Gastroesophageal Reflux Disease (FSSG), showed significant improvement within the first week of treatment and sustained benefits over 28 days (*p* < 0.05 for all time points compared to baseline). Following the initial treatment phase, 21 patients were subjected to a maintenance regimen of vonoprazan 10 mg daily after breakfast for 8 weeks. At the end of the maintenance phase, in 76.2% (16/21) of the patients at gastroscopy, no signs of esophagitis were shown, which documents the vonoprazan’s efficacy in both short-term and long-term treatment of PPI-resistant RE [51].

A smaller prospective study involved eight patients with persistent gastric mucosal injury after an 8-week course of standard PPI therapy (rabeprazole 10 mg/day, esomeprazole 20 mg/day, or lansoprazole 30 mg/day in the morning) and aimed to evaluate the efficacy of vonoprazan in treating gastroesophageal reflux disease (GERD) refractory to proton pump inhibitor (PPI) therapy. All the patients were extensive metabolizers of CYP2C19. Six patients were diagnosed with ineffective esophageal motility, all of whom had a hiatal hernia [52].

The patients were assessed using high-resolution manometry (HRM) and 24 h MII-pH monitoring while on PPI therapy as baseline measurements. After completing the initial tests, the patients received 20 mg of vonoprazan daily for 4 weeks. Following this treatment period, the participants underwent an upper endoscopy and 24 h MII-pH monitoring. pH was monitored 5 cm above and 10 cm below the proximal edge of the LES [52].

The result showed that 87.5% (7 out of 8) patients achieved complete mucosal lesions healing, characterized by the resolution of visible lesions, as confirmed by follow-up endoscopy after 4 weeks of vonoprazan treatment. The patient with Los Angeles (LA) grade C esophagitis did not achieve complete mucosal healing after 4 weeks of vonoprazan therapy, with persistent lesions (grade A) remaining. The patient continued vonoprazan therapy at 20 mg daily for an additional 4 weeks, ultimately achieving complete mucosal healing [52].

Additionally, gastric pH control improved significantly, with the median 24 h gastric pH > 4 HTR increasing from 26.5% during PPI therapy to 78.0% after four weeks of vonoprazan treatment, suggesting superior gastric acid control with vonoprazan compared to PPIs [52]. Moreover, nocturnal gastric acid breakthrough decreased from 87.5% (7 z 8) of the patients during PPI therapy to 50% after switching to vonoprazan, highlighting superior gastric acid control with vonoprazan compared to PPIs. Additionally, four of the eight patients experienced complete resolution of reflux symptoms (heartburn or regurgitation) after switching to vonoprazan [52].

It should be emphasized that the doses of proton pump inhibitors (PPIs) used in some studies—such as esomeprazole 20 mg daily—were lower than the standard doses typically recommended for patients with refractory GERD. For optimal acid suppression, increasing the PPI dose (e.g., to esomeprazole 40 mg daily) or switching to a different PPI may be necessary. These factors should be considered when interpreting efficacy comparisons between PPIs and P-CABs such as vonoprazan [4].

#### 3.4.2. Efficacy in GERD Symptom Control and Long-Term Healing

In a retrospective analysis of 55 GERD patients (30 with non-erosive reflux disease [NERD] and 25 with erosive esophagitis [EE]), the effect of daily oral vonoprazan 10 mg was evaluated over the course of one year. Symptom assessment was performed using the Izumo scale, a validated questionnaire for gastrointestinal symptoms. After one month of treatment, 89% of the patients reported significant improvement in symptoms, including heartburn, regurgitation, throat discomfort, postprandial distress, epigastric pain, constipation, and diarrhea. Additionally, symptom improvement was statistically significant at all time points during the one-year follow-up, as assessed by the Izumo scale (*p* < 0.001). Furthermore, 82% of these patients maintained this improvement throughout the entire year of vonoprazan therapy. Among all the patients who used the treatment for one year, 47% (26/55) reported sustained resolution and long-term relief from GERD symptoms. Notably, 95% of the patients with erosive esophagitis had maintained endoscopic healing in a follow-up endoscopy conducted at 12 months [53].

In a similar retrospective study involving 88 GERD patients treated with 10 mg of vonoprazan daily, 86% experienced symptom improvement, including reductions in heartburn, regurgitation, throat discomfort, epigastric pain, postprandial distress, constipation, and diarrhea, with 57% achieving complete resolution of symptoms within one month. The patients with erosive esophagitis demonstrated a significantly higher rate of symptom resolution (71%) compared to those with non-erosive disease (47%) (*p* = 0.025). The observed difference in treatment response may be attributed to the heterogeneous pathophysiology of NERD. NERD encompasses a wide range of underlying mechanisms, including esophageal hypersensitivity, functional heartburn, esophageal motility disorders, and both acid and non-acid reflux. Furthermore, impaired esophageal motility, which leads to delayed acid clearance, may further contribute to the persistence of symptoms despite adequate acid suppression. These factors underscore the necessity for individualized diagnostic approaches and tailored therapeutic strategies before considering the escalation to more aggressive treatment regimens [54].

According to the results of the multivariate analysis conducted by the authors, older age (≥60 years, *p* = 0.002), obesity (BMI ≥ 24, *p* = 0.030), and erosive esophagitis (*p* = 0.018) were identified as strong predictors of a better response to vonoprazan treatment [43]. Older patients may have better compliance and reduced gastric sensitivity and/or motility, which contributes to better treatment outcomes [55]. Obesity is associated with increased gastric acid secretion and higher visceral fat, which stimulate gastrin release and contribute to acid reflux. This increased acid production in obese patients makes their GERD symptoms more responsive to acid suppression therapies, as reducing excess acid directly alleviates their symptoms [54,56]. In contrast, alcohol consumption (*p* = 0.011) and a history of Helicobacter pylori eradication (*p* = 0.015) were found to be negative predictors. Alcohol (>20 g daily) can directly damage the esophageal mucosa, impair motility by slowing normal peristalsis, and lower LES pressure, which decreases the efficacy of acid suppression therapies by making it easier for stomach acid to flow back into the esophagus [57]. In patients with a history of *Helicobacter pylori* eradication, atrophic changes in the gastric lining can reduce acid secretion, further limiting the effectiveness of acid-suppressive therapies [58].

In a prospective, randomized, double-blind trial involving 32 patients with erosive esophagitis and heartburn occurring at least once a week, patients were randomly assigned to receive either 20 mg of vonoprazan or 30 mg of lansoprazole daily before breakfast for 14 days. The study aimed to assess the onset and duration of symptom relief, with a particular focus on heartburn [59].

By the first day of treatment, 37.5% (6/16) of the patients treated with vonoprazan experienced complete relief from their heartburn symptoms during the day for the next 7 consecutive days compared to only 18.8% (3/16) of the patients receiving lansoprazole. The difference in the onset of daytime heartburn relief between the two groups was statistically significant (*p* < 0.05). The differences in relief were even more pronounced for nocturnal heartburn. On Day 1, 33.3% (5/15) of the patients treated with vonoprazan achieved complete relief from nocturnal heartburn for the following 7 days, whereas only 9.1% (1/11) of the patients receiving lansoprazole reported similar results, with the difference reaching statistical significance (*p* < 0.01). These findings underscore vonoprazan’s superior efficacy in alleviating heartburn symptoms, particularly nocturnal symptoms, which are often more difficult to manage with traditional proton pump inhibitors like lansoprazole [59].

In addition to its faster and more sustained relief of heartburn symptoms, vonoprazan also demonstrated significant improvements in sleep quality compared to lansoprazole. After 14 days of treatment, sleep quality, as assessed by the Pittsburgh Sleep Quality Index (PSQI), showed a significant improvement in patients treated with vonoprazan (*p* < 0.05), while no notable change was observed in patients receiving lansoprazole [59].

In a subsequent, randomized, double-blind, phase III trial, healing rates at 8 weeks were similar between vonoprazan 20 mg (92.4%) and lansoprazole 30 mg (91.3%) among 468 Asian patients with erosive esophagitis. Vonoprazan demonstrated numerically greater early efficacy, with 75.0% healing after 2 weeks compared to 67.8% for lansoprazole. Although these early differences did not reach statistical significance, vonoprazan showed better outcomes, particularly in patients with more severe disease (Los Angeles grades C/D). Importantly, adverse event rates were comparable between the groups (38.1% vs. 36.6%), supporting a similar safety profile [60].

A prospective, randomized, double-blind controlled trial (RCT) by Laine et al. (2023) also confirmed vonoprazan’s 20 mg superiority in the early phases of treatment for erosive esophagitis [61]. After 2 weeks, vonoprazan showed significantly better healing rates, defined as endoscopically confirmed resolution of erosive lesions, in patients with grade C/D esophagitis (70.2% for vonoprazan vs. 52.6% for lansoprazole 30 mg; *p* = 0.0008) based on a result from 1024 participants. Additionally, heartburn relief in the maintenance phase, was similar between the two groups, with 80.6% of the patients on vonoprazan (20 mg) reporting 24 h heartburn-free days compared to 78.6% in the lansoprazole group (15 mg) based on the findings from 878 patients. The observed difference of 2.0% (95% CI: −2.6 to 6.7) did not reach statistical significance for superiority. However, non-inferiority was established, as the lower bound of the confidence interval was above the predefined margin of −15%.

In a randomized, multicenter clinical trial evaluating on-demand vonoprazan therapy for non-erosive reflux disease (NERD), vonoprazan demonstrated significantly higher efficacy in relieving heartburn compared to placebo. In the groups receiving vonoprazan (10 mg, 20 mg, and 40 mg), a significantly higher percentage of heartburn episodes were completely relieved within 3 h of dosing, with relief lasting for 24 h. Heartburn episodes were measured using daily diaries, where patients recorded the onset and intensity of symptoms. These episodes were then assessed for complete relief within 3 h and sustained relief for 24 h. The on-demand therapy lasted for 6 weeks, during which the patients took vonoprazan only when experiencing heartburn symptoms: with 10 mg vonoprazan, 56% of the heartburn episodes were fully relieved, with 20 mg—60.6%, and with 40 mg—70%. In contrast, only 27.3% of the heartburn episodes were completely relieved in the placebo group (*p* < 0.0001). Vonoprazan also demonstrated significantly higher efficacy within the first hour post-dose compared to the placebo (*p* < 0.0001) [62].

A 2024 meta-analysis by Zhuang et al. compared the efficacy and safety of vonoprazan and proton pump inhibitors (PPIs) in treating Los Angeles grade C/D esophagitis [63]. The analysis included 24 randomized controlled trials (RCTs) comparing three P-CABs (vonoprazan, tegoprazan, and keverprazan) and six PPIs (lansoprazole, esomeprazole, omeprazole, rabeprazole extended-release, pantoprazole, and dexlansoprazole). Surface Under the Cumulative Ranking Curve (SUCRA) scores were used to assess the relative efficacy of these treatments. SUCRA is a statistical measure used in meta-analysis to rank interventions based on their likelihood of being the most effective. A higher SUCRA score indicates a greater likelihood of a treatment being ranked as the best option [63].

For initial treatment, vonoprazan 20 mg/day demonstrated a significantly lower risk of treatment failure compared to 30 mg/day of lansoprazole (RR = 2.72, 95% CI [1.10–6.73], *p* = 0.03) and 20 mg/day of omeprazole (RR = 3.51, 95% CrI [1.31–12.14]). Significant superiority was observed specifically against lansoprazole and omeprazole, while comparisons with other PPIs did not show significant differences. Vonoprazan 20 mg/day reduced the absolute risk of treatment failure by 11–21% compared to PPIs, with treatment failure rates of 6% for vonoprazan versus 21% for overall PPIs. The ranking analysis confirmed that vonoprazan 20 mg q.d. (SUCRA = 0.89) was the top-performing treatment for grade C/D esophagitis, far outpacing all the PPI and P-CAB options. For instance, kaverprazan 20 mg/day ranked second with a SUCRA score of 0.87, followed by dexlansoprazole 60 mg/day (SUCRA = 0.66) and esomeprazole 40 mg/day (SUCRA = 0.61). For maintenance treatment, direct comparisons showed the superiority of vonoprazan 20 mg/day over lansoprazole 15 mg/day (RR = 8.39, 95% CI [2.06–34.24], *p* = 0.001), but pooled network analysis did not demonstrate significant differences between vonoprazan and other PPIs. Nevertheless, vonoprazan 20 mg/day reduced the absolute risk of treatment failure by 4–18% compared to PPIs, with an estimated treatment failure rate of 16% for vonoprazan compared to 30% for other PPIs [63].

Vonoprazan exhibited a favorable safety profile, with similar rates of adverse events (AEs), serious adverse events (SAEs), and drug withdrawal compared to PPIs. In short-term safety rankings, vonoprazan 20 mg q.d. (SUCRA = 0.59) performed better than 40 mg of esomeprazole q.d. (SUCRA = 0.42) and 60 mg of dexlansoprazole q.d. (SUCRA = 0.52), further confirming its relative safety in comparison to maximized-dose PPIs [63].

Considering both efficacy and safety outcomes, 20 mg of vonoprazan q.d. demonstrated superior effectiveness and was relatively safe in the initial treatment of grade C/D esophagitis [63].

In the 5-year VISION study, which aimed to evaluate the long-term efficacy and safety of vonoprazan, the results demonstrated that vonoprazan provided superior long-term control of recurrent erosive esophagitis (EE) compared to lansoprazole. The cumulative recurrence rate over 260 weeks was 10.8% (95% CI, 6.4–18.0%) for vonoprazan and 38.0% (95% CI, 25.5–54.1%) for lansoprazole (*p* = 0.001). While initial healing rates were similar (96.4% for vonoprazan vs. 97.1% for lansoprazole, *p* = 1.00), vonoprazan showed significantly better prevention of recurrence during long-term maintenance therapy [64].

Although lifestyle modifications such as avoiding meals 2–3 h before bedtime, elevating the head of the bed, and weight reduction in overweight or obese patients are recommended for GERD management, these strategies were not consistently evaluated in most studies reviewed [2]. Some studies did include fixed mealtimes and postural modifications, but these were not the primary focus [52]. Additionally, reducing alcohol consumption and quitting smoking may also benefit patients with GERD. The limited reporting of non-pharmacologic strategies may restrict the interpretation of treatment resistance, highlighting the need for more comprehensive assessment in future studies [2,4].

Table 2, Table 3 and Table 4 summarize the clinical efficacy, patient outcomes, and meta-analytical findings regarding vonoprazan use in GERD management.

### 3.5. Efficacy of Tegoprazan in the Treatment of Erosive Esophagitis and Non-Erosive Reflux Disease

In a 2025 study conducted by Kang et al., the efficacy of on-demand therapy with tegoprazan was evaluated in patients with GERD, including both NERD and EE. This was a randomized, open-label study involving 76 adult patients with a history of heartburn and regurgitation. All the participants had previously undergone acid-suppressive therapy (PPI once daily) for at least four weeks in the case of NERD and eight weeks for EE, with documented symptom improvement. The patients were then randomly assigned to receive either tegoprazan (50 mg) or esomeprazole (20 mg) on an as-needed basis for eight weeks [65].

The results showed that tegoprazan provided faster symptom relief compared to esomeprazole. Among all the episodes of heartburn and regurgitation, 26.2% resolved within 30 min after taking tegoprazan versus 16.1% after esomeprazole (*p* < 0.05), with sustained symptom relief for 24 h. This significant difference in rapid symptom improvement was consistently observed up to three hours post-administration (*p* < 0.05 at each time point) [65].

During the first four weeks, the number of doses taken was similar in both groups (mean (SD): 10.64 (7.96) for tegoprazan vs. 10.15 (10.52) for esomeprazole), with median values of 10 and 7, respectively. However, in the following four weeks, the patients using tegoprazan required fewer doses (mean [SD]: 9.47 (7.92) vs. 10.67 (9.23)), with median values of 7 and 8, respectively, suggesting better long-term symptom control [65].

In a randomized, multicenter, double-blind trial, researchers evaluated the effectiveness of tegoprazan (50 mg) compared to lansoprazole (30 mg) in the treatment of EE. A total of 218 patients with endoscopically confirmed EE (Los Angeles Classification Grades A–D) were randomly assigned to receive either tegoprazan or lansoprazole once daily. The primary endpoint was the cumulative proportion of patients achieving mucosal healing as confirmed by endoscopy by week 4, while secondary endpoints included healing rates at week 2 [66].

The analysis included 103 patients in the tegoprazan group and 109 in the lansoprazole group. By week 4, the cumulative healing rate was 95.2% (98/103) for tegoprazan and 86.2% (94/109) for lansoprazole, with a statistically significant difference of 8.91% (95% confidence interval [CI]: 1.22–16.59; *p* < 0.0001 for non-inferiority and 0.0266 for superiority). At week 2, healing rates were 88.4% (91/103) for tegoprazan and 82.6% (90/109) for lansoprazole, with a difference of 5.78% (95% CI: −3.66–15.22; *p* = 0.0005 for non-inferiority) [66].

In a randomized, double-blind, placebo-controlled, multicenter study, 324 patients diagnosed with NERD were assigned to one of three treatment groups: tegoprazan 50 mg, tegoprazan 100 mg, or placebo. Each treatment was administered once daily for a duration of 4 weeks. The primary outcome of the study was the proportion of patients achieving complete resolution of the major symptoms (heartburn and regurgitation) during the last 7 days of the treatment period [67].

In the study, 42.5% of the patients in the tegoprazan 50 mg group, 48.5% in the tegoprazan 100 mg group, and 24.2% in the placebo group achieved complete resolution of heartburn and regurgitation by week 4. Both doses of tegoprazan demonstrated significantly greater efficacy compared to placebo (*p* < 0.05 for both comparisons). The resolution rates for heartburn, as well as the proportion of heartburn-free days, were significantly higher in both tegoprazan groups than in the placebo group (*p* < 0.05). Specifically, 62.3% of the patients receiving tegoprazan 50 mg and 65.7% receiving tegoprazan 100 mg achieved complete resolution of heartburn compared to 43.4% in the placebo group by week 4 [67].

Table 5 summarizes the clinical outcomes of tegoprazan therapy in patients with erosive esophagitis and non-erosive reflux disease.

### 3.6. Safety of Potassium-Competitive Acid Blockers (PCABs)

#### 3.6.1. Short-Term Safety Profile of Vonoprazan

The short-term safety profile of vonoprazan is comparable to that of proton pump inhibitors (PPIs). Approximately 20% of the patients experience adverse events (AEs), with 7% being drug-related, 1% classified as serious AEs, and 1% leading to treatment discontinuation. Nearly all the reported adverse events (AEs) are mild in short-term use, and the overall safety of vonoprazan is like that of PPIs [68].

According to the integrated safety data analysis of vonoprazan, which included 14 clinical trials and post-marketing surveillance, the most frequently observed adverse events included, among others, nasopharyngitis (6.94%), diarrhea (2.69%), upper respiratory tract infections (1.96%), gastroenteritis (1.43%), nausea (1.35%), constipation (1.20%), abdominal distension (1.17%), and upper abdominal pain (1.13%). Other common adverse events included headache (1.00%) and dizziness (0.88%). For comparison, lansoprazole and esomeprazole showed a similar frequency of certain adverse events, such as nasopharyngitis (5.07%), diarrhea (3.68%), upper respiratory tract infections (2.83%), nausea (0.99%), headaches (1.74%), and constipation (1.19%) [69].

Adverse event reports submitted to the Japanese Pharmaceuticals and Medical Devices Agency between 2004 and 2017 were analyzed in a retrospective pharmacovigilance study using the JADER (Japanese Adverse Drug Event Report) database. The study included 11,433 reports related to PPIs and 636 reports related to vonoprazan [70].

The most common adverse events linked to proton pump inhibitors (PPIs) were abnormal liver function, interstitial lung disease, microscopic colitis, and agranulocytosis. For vonoprazan, the most frequent events included drug eruption, abnormal hepatic function, hemorrhagic enterocolitis, and rash [70].

Both vonoprazan and PPIs were found to be associated with hepatotoxicity, skin reactions (such as toxic epidermal necrolysis and erythema multiforme), and pancytopenia. No significant association was found between vonoprazan and interstitial lung disease, which was strongly linked to PPIs. In contrast, hemorrhagic enterocolitis was particularly associated with vonoprazan, suggesting that this adverse event is more characteristic of vonoprazan than of PPIs. Lansoprazole showed a marked tendency to cause microscopic colitis. Both PPIs and vonoprazan were similarly associated with pancytopenia, highlighting the importance of careful monitoring for hematologic adverse effects when initiating treatment with either drug [70].

Table 6 summarizes selected adverse events reported with vonoprazan and PPIs based on clinical trials and pharmacovigilance data.

#### 3.6.2. Long-Term Safety of PCABs: The VISION Study

The VISION study is the first to assess the long-term safety of vonoprazan in Japanese patients with healed erosive esophagitis (EE). It evaluated the safety and efficacy of vonoprazan compared to lansoprazole, with a focus on gastric mucosal changes and the potential for malignant transformations. Patients were initially treated with vonoprazan (20 mg daily) or lansoprazole (30 mg daily) for up to 8 weeks to heal EE. A total of 202 patients were randomly assigned to maintenance therapy with either vonoprazan (10 mg daily) or lansoprazole (15 mg daily) for a total of 260 weeks (5 years) [64].

No cases of neuroendocrine tumors (NETs) or malignant epithelial transformations were observed in either group. Two cases of adenomas were reported: one case of foveolar-type adenoma in the vonoprazan group after 3 years of therapy and one case of oxyntic gland adenoma in the lansoprazole group after 4 years. Neither lesion was malignant [64]. However, histopathological changes, including parietal cell protrusion and foveolar hyperplasia, were more frequent in the vonoprazan group, especially after five years of therapy. The prevalence of parietal cell hyperplasia was significantly higher in the vonoprazan group compared to the lansoprazole group at weeks 48, 108, 156, and 260 (97.1% vs. 86.5% at week 260). At week 260, more patients in the vonoprazan group had foveolar hyperplasia (14.7%) compared to the lansoprazole group (1.9%), with both groups remaining similar until week 204, with rates below 10% in both groups prior to that time. ECL cell hyperplasia remained low throughout the study period (<10%), with rates of 4.9% in the vonoprazan group and 7.7% in the lansoprazole group at week 260. At earlier time points (e.g., weeks 48 and 108), the differences in ECL cell hyperplasia between the two groups were minimal. At week 260, a higher proportion of patients in the vonoprazan group had G-cell hyperplasia (85.3%) compared to the lansoprazole group (76.9%). Throughout the entire study period, the incidence of G-cell hyperplasia was higher in the vonoprazan group [64].

Serum gastrin and chromogranin A levels were consistently higher in the vonoprazan group at every time point throughout the entire study, starting from week 4 of the healing phase and continuing at all the subsequent assessments during the maintenance phase. The median serum gastrin level at week 260 was 625 pg/mL in the vonoprazan group and 200 pg/mL in the lansoprazole group. Similarly, the median serum chromogranin A levels at week 260 were 250 ng/mL for vonoprazan compared to 100 ng/mL for lansoprazole [64].

Adverse events (AEs) were reported in both treatment groups, with the overall incidence of AEs being similar between the two groups during the entire maintenance phase. The most common AEs were mild gastrointestinal disorders, with gastric polyps being the most frequently observed, occurring in 45.9% of the patients in the vonoprazan group and 46.3% in the lansoprazole group. Nasopharyngitis was reported in 34.1% of the patients receiving vonoprazan and 43.3% of those on lansoprazole. Large intestine polyps were observed in 11.9% and 13.4% of the patients, respectively. Other frequently reported AEs included eczema (6.7% in the vonoprazan group vs. 14.9% in the lansoprazole group), hypertension (11.0% vs. 9.0%), erosive gastritis (8.9% vs. 13.4%), and bronchitis (8.9% vs. 10.4%) [64].

Additionally, AEs related to diarrhea occurred in 8.9% of the patients in the vonoprazan group and 6.0% in the lansoprazole group, while those associated with bone fractures were noted in 8.1% and 11.9%, respectively. Pneumonia-related AEs were infrequent, affecting 1.5% of the patients in both groups. Dementia-related AEs were also uncommon, with 0.7% in the vonoprazan group and 1.5% in the lansoprazole group [64]. Although the VISION study offers preliminary insights into the long-term safety of vonoprazan, including data on fracture incidence, it does not provide a direct evaluation of bone mineral density or vitamin B12 levels. Given that both osteoporosis and B12 deficiency may develop gradually with prolonged acid suppression, further long-term studies focused on these outcomes are needed. Addressing this gap is essential to fully understand the long-term safety of vonoprazan and other PCABs [64].

In terms of serious adverse events, only 2.2% of the patients in the vonoprazan group (3 patients) experienced serious AEs that were considered related to treatment, including leukopenia, acute cholangitis, and abnormal liver function. In contrast, no serious AEs related to treatment were reported in the lansoprazole group [64].

Regarding treatment discontinuation due to AEs, six patients (4.4%) in the vonoprazan group and one patient (1.5%) in the lansoprazole group discontinued therapy. The reasons for discontinuation in the vonoprazan group included abnormal liver function, acute cholangitis, duodenal obstruction, leukopenia, loss of consciousness, and pain, while in the lansoprazole group, discontinuation occurred in only one patient (1.5%), with a single case of thrombotic cerebral infarction [64].

Although vonoprazan is highly effective in suppressing gastric acid production, concerns have been raised about the potential for rebound acid hypersecretion following its abrupt discontinuation, possibly due to elevated serum gastrin levels observed during therapy. However, available clinical data remain limited. In a study simulating the transition from daily vonoprazan to on-demand therapy after symptom control, no evidence of increased reflux symptoms or rebound acid secretion was observed, suggesting that abrupt discontinuation may not necessarily lead to clinically significant worsening of symptoms [62].

#### 3.6.3. Impact on Intestinal Microbiota and Infections

The impact of proton pump inhibitors (PPIs) and potassium-competitive acid blockers (PCABs) on the gut microbiota is an important consideration, as these medications can influence the risk of infections, such as Clostridioides difficile. In a study where healthy, Helicobacter pylori-negative individuals were treated with either vonoprazan (20 mg daily) or lansoprazole (30 mg daily) for 4 weeks, fecal samples were collected before and after treatment, and microbiota changes were analyzed using 16S rRNA gene sequencing [71]. The results revealed that vonoprazan caused more pronounced changes in the gut microbiome compared to lansoprazole. Vonoprazan led to a significant increase in the abundance of genera such as Actinomyces, Rothia, Granulicatella, Bacteroides, and Streptococcus. Notably, it also decreased the abundance of beneficial genera like Blautia and Coprococcus, which are known for their protective role against Clostridioides difficile infection. Coprococcus plays a crucial role in the gut microbiota, particularly as a producer of butyric acid [72]. Butyric acid, a primary component of short-chain fatty acids, has been shown to help prevent the translocation of C. difficile and alleviate inflammation caused by this bacterium. It does so by strengthening the tight junctions between intestinal epithelial cells, which reduces intestinal permeability and enhances protection against infection [73]. In contrast, lansoprazole treatment primarily promoted an increase in Bacteroides and Streptococcus species, with fewer significant changes in other genera [71].

An increase in Bacteroides can disrupt the balance of the intestinal microbiota and, when it overgrows, may lead to infections outside the gastrointestinal tract. It has been observed that both vonoprazan and proton pump inhibitors (PPIs) decrease the abundance of Bifidobacterium, a genus essential for maintaining the intestinal barrier. Bifidobacterium helps protect the gut by forming a barrier against harmful bacteria like Escherichia coli and supporting gut microbiota balance. Its decreased abundance may compromise gut health, further increasing intestinal permeability and contributing to diarrhea [74].

**Figure 1:**
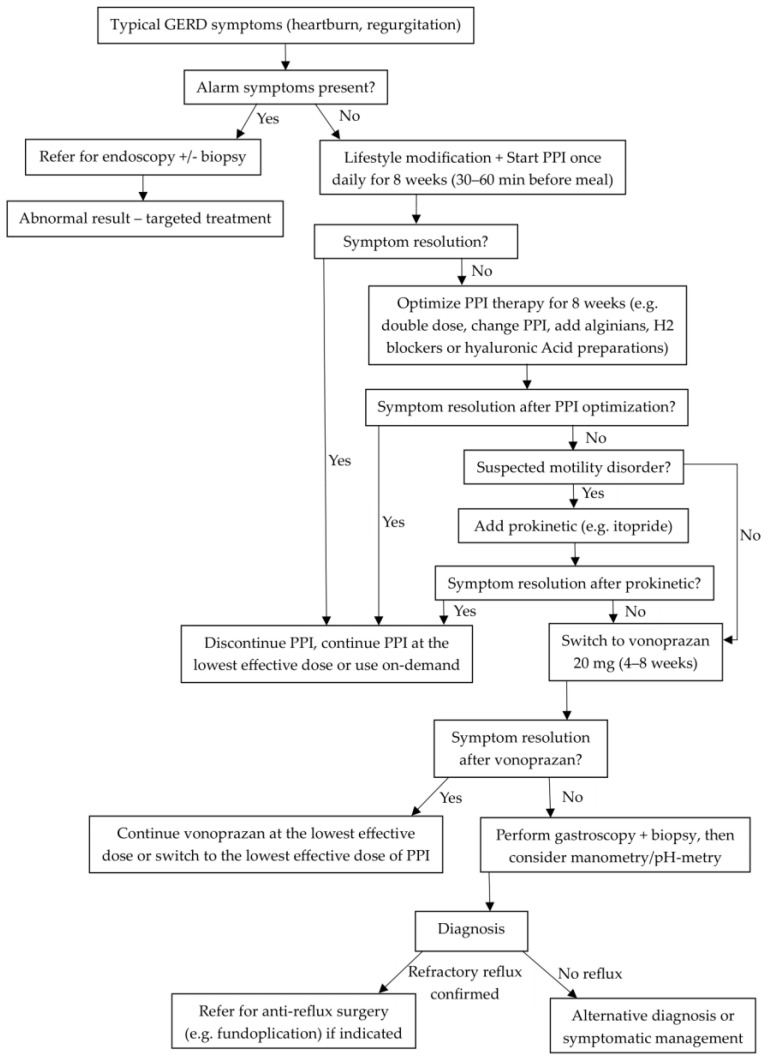
Simplified treatment algorithm for GERD, based on current international guidelines. This figure represents the authors’ clinical interpretation and positioning of PCABs alongside PPIs in GERD management [4,13,14,15].

To conclude the section on PCABs, Figure 1 presents a simplified treatment algorithm for GERD proposed by the authors to illustrate the clinical positioning of PCABs in line with the current guidelines.

## 4. The Role of Prokinetic Therapy in GERD Management

Itopride, a prokinetic agent, dopamine D2 receptor antagonist, and acetylcholinesterase inhibitor, has gained attention for its potential in treating GERD. Reflux disease is not solely the result of excessive acid production; various functional abnormalities of the esophagus and stomach, including impaired esophageal clearance due to weak peristalsis and low-amplitude contractions in the distal esophagus, as well as delayed gastric emptying, may also contribute significantly to its pathogenesis [75,76]. These mechanisms can lead to the delayed removal of refluxed acid from the esophagus, thereby prolonging acid exposure and exacerbating symptoms. By targeting these motility-related abnormalities, prokinetic therapy may enhance acid clearance, support standard PPI treatment, and contribute to more effective symptom control [75].

By enhancing the contraction of smooth muscle, increasing gastrointestinal motility, strengthening lower esophageal sphincter (LES) tone, and accelerating gastric emptying, itopride appears to be a potentially therapeutic option, particularly for patients with functional dyspepsia or delayed gastric emptying [77]. In patients with pharmacologically refractory GERD, high-resolution esophageal manometry should be performed to evaluate esophageal motility disorders and guide further management. In selected cases, additional gastric emptying studies, such as scintigraphy, may also be considered. Moreover, patients with laryngopharyngeal reflux, which can involve persistent throat symptoms such as heartburn, grunting, coughing, and throat obstruction, may benefit from prokinetic therapy. Prokinetic therapy may, thus, improve overall treatment outcomes in selected patient groups [76,78]. Among available prokinetics, agents such as itopride and mosapride have been investigated for their potential role in GERD management. Mosapride, a selective 5-HT4 receptor agonist, enhances acetylcholine release at enteric nerve endings and promotes gastrointestinal motility; its active metabolite (M1) additionally acts as a 5-HT3 receptor antagonist, potentially supporting its prokinetic effects [79].

One significant advantage of itopride is its inability to cross the blood–brain barrier, and its use is not associated with the extrapyramidal symptoms, dyskinesias, or neuroleptic syndrome observed with other prokinetic agents. In addition, itopride does not prolong the QT interval, further confirming its safety profile. It is generally well tolerated by patients, with side effects being rare and mild. These may include gastrointestinal disorders: diarrhea and abdominal pain. Some patients may experience headaches or a slight increase in prolactin levels, though these occurrences are uncommon. Itopride’s metabolism is independent of the cytochrome P450 system, reducing the risk of drug interactions [77].

A recent retrospective study evaluated the efficacy and safety of combining itopride with PPIs in the treatment of GERD, and showed a statistically significant reduction in symptoms when this combination therapy was applied compared to the use of PPIs alone. The study included 140 patients with typical GERD symptoms, despite at least 8 weeks of prior IPP therapy twice daily. The diagnosis of GERD was confirmed via 24 h esophageal impedance-pH monitoring. The daily dose of itopride was 150 mg, administered in three divided 50 mg doses, 30 min before meals, for 8 weeks. The greatest improvement was observed in symptoms related to acid overproduction (heartburn, stomach, and esophageal burning) and motility disturbances (dysphagia, postprandial fullness, gastric retention, and nausea), as well as in laryngopharyngeal symptoms. Itopride demonstrated a favorable safety profile, with no notable drug interactions or serious side effects that could lead to complications [75].

In another comparative, prospective study, combination therapy proved more effective in relieving GERD symptoms compared to pantoprazole monotherapy. The study included 100 patients who were randomly assigned to two groups. In the first group, patients received pantoprazole 40 mg twice daily, while in the second group, itopride 50 mg three times daily, 30 min before meals, was added to the regimen. The results for the endoscopic healing of esophagitis were similar in both groups, with 72% of the patients in the group receiving pantoprazole alone and 74% in the group receiving combination therapy. However, symptom reduction was significantly greater in the combination therapy group (74.5%) compared to the pantoprazole-only group (62.5%). Adverse effects (nausea, abdominal pain, diarrhea, and headache) were more frequently observed in the pantoprazole-only group (22% vs. 30%). These findings suggest that the addition of itopride to standard PPI therapy offers a promising approach for enhancing symptom relief, with a favorable safety profile [80].

## 5. Future Directions in GERD Treatment

IW-3718, a novel bile acid sequestrant (colesewelam) with gastric retention technology (Acuform), is showing potential as an adjunct to proton pump inhibitors (PPIs) for the treatment of refractory gastroesophageal reflux disease (GERD). In a randomized, double-blind, placebo-controlled trial involving 280 patients with gastroesophageal reflux disease using standard doses of PPIs, the addition of 1500 mg of IW-3718 twice daily significantly reduced the severity of heartburn by 58% compared to a 46% reduction in the placebo group. IW-3718 was generally well tolerated, with constipation, flatulence, nausea, and upper respiratory tract infections being the most common side effects [81].

The role of duodeno-gastro-esophageal reflux (DGER), in which bile acids reflux from the duodenum into the esophagus, is considered an important cause of persistent GERD symptoms, particularly in treatment-resistant cases. Bile salts can damage the esophageal mucosa by increasing mucosal permeability and inducing dilated intercellular spaces [82,83,84]. DGER has also been associated with more advanced forms of mucosal injury, including erosive esophagitis and Barrett’s esophagus [85,86]. Importantly, up to 65% of the patients with persistent symptoms despite PPI therapy may have clinically significant DGER. This provides a strong rationale for incorporating bile acid-targeted therapies, such as IW-3718, into treatment strategies for refractory GERD [81,87].

Due to the clinical overlap between acid and non-acid reflux, symptoms alone are insufficient to distinguish between these subtypes. Therefore, an accurate diagnosis of non-acid reflux requires the use of combined 24 h pH-impedance monitoring (pH-MII). However, limitations remain as the interpretation of impedance tracings is labor-intensive, consensus on normal thresholds is lacking, and symptom-reflux correlations may be missed during short monitoring windows. Despite these challenges, pH-MII remains essential in identifying patients in whom non-acid reflux may be a major contributor to symptom persistence [88].

Since bile acid reflux has been implicated in the pathophysiology of GERD, particularly in cases where the acidic component of reflux is diminished, IW-3718’s mechanism of action—binding bile acids in the stomach and preventing their entry into the esophagus—may help alleviate esophageal injury and reduce symptom exacerbation. By mitigating the harmful effects of bile acid reflux, IW-3718 could improve symptom control and provide significant relief for patients who do not respond adequately to PPI therapy alone [81,89].

Looking ahead, further research into the use of IW-3718 in combination with other emerging therapies, such as prokinetic agents targeting different reflux mechanisms, could open new therapeutic avenues for GERD management. Additionally, optimizing dosing regimens to balance efficacy with minimal side effects will be crucial for improving patient adherence to treatment. Expanding studies to assess IW-3718’s potential in specific subgroups of GERD patients—such as those with Barrett’s esophagus or erosive esophagitis—could identify additional clinical indications for its use [81].

## 6. Conclusions

Recent advancements in the treatment of gastroesophageal reflux disease (GERD) have led to the emergence of several promising therapeutic options that extend beyond traditional proton pump inhibitors (PPIs).

Potassium-competitive acid blockers (PCABs), such as vonoprazan and tegoprazan, have shown significant promise in both the short- and long-term management of GERD. PCABs offer superior acid suppression compared to PPIs, particularly in patients with erosive esophagitis. Vonoprazan has demonstrated better control of recurrent erosive esophagitis during maintenance therapy, with significantly lower recurrence rates compared to PPIs. PCABs have also shown greater efficacy in more severe or advanced cases of erosive esophagitis (EE), making them a valuable option for patients with difficult-to-treat or refractory diseases. However, long-term monitoring remains essential due to the potential for adverse effects such as hypergastrinemia, gastric polyps, and parietal cell hyperplasia. The role of on-demand therapy with PCABs is being investigated, offering potential benefits for patients with intermittent symptoms who may not require continuous medication.

Prokinetic agents like itopride are gaining attention as adjunctive therapies for GERD, particularly for patients with motility disorders or functional dyspepsia. Itopride works by accelerating gastric emptying without the neurological side effects common to older prokinetic agents. Studies have shown that combining itopride with PPIs provides better symptom relief, especially in patients struggling with regurgitation and postprandial discomfort. The absence of significant drug interactions and its favorable safety profile make itopride an appealing option for managing GERD symptoms that extend beyond acid suppression.

IW-3718, a novel bile acid sequestrant, has demonstrated efficacy as an adjunctive therapy for refractory GERD, especially in patients with bile acid reflux. By binding bile acids in the stomach and preventing their reflux into the esophagus, IW-3718 reduces heartburn severity and improves symptom control in patients who do not respond adequately to PPIs. Its potential for use in specific subgroups, such as those with Barrett’s esophagus or erosive esophagitis, suggests promising benefits, though further evaluation of optimal dosing and long-term safety is needed.

While PPIs remain the cornerstone of treatment, the emergence of alternative pharmacologic options marks a new era in GERD management, offering hope for patients with refractory or complex disease presentations. Continued clinical trials, real-world studies, and long-term safety evaluations will further refine treatment algorithms and improve patient outcomes in GERD therapy.

## Figures and Tables

**Table 1:** Comparison of proton pump inhibitors (PPIs) and potassium-competitive acid blockers (P-CABs) [7,40,47].

Characteristic	Proton Pump Inhibitors (PPIs)	Potassium-Competitive Acid Blockers (P-CABs)
Prodrug Requirement	Yes, requires transformation to the active form (sulfenamide)	No, acts directly on H^+^/K^+^-ATPase after protonation
Binding Site	Binds covalently to the H^+^/K^+^-ATPase	Binds competitively to the K^+^-binding site of H^+^/K^+^-ATPase
Binding Type	Irreversible binding to the proton pump	Reversible binding to the proton pump
Onset of Full Effect	After 3–5 days	After the first dose
Genetic Polymorphism Impact	Affected (influenced by CYP2C19 polymorphism)	Not affected
Pharmacodynamic Effect	More significant during the daytime	Lasts throughout both daytime and nighttime
Antisecretory Activity	Meal-dependent	Meal-independent
Half-Life	1–2 h	7–9 h (vonoprazan)
Optimal Dosing Administration	30–60 min before meals (for most PPIs)	Independent of mealtime (before or after meals)

**Table 2:** Clinical efficacy of vonoprazan in the treatment of erosive esophagitis [59,60,61].

Drug Name	Patient Group	Esophageal Response Rate	Extraesophageal Response Rate	Evaluation Method	Drug Dose	Treatment Duration	Reference Number
Vonoprazan vs. Lansoprazole	RE	Healing at 2 weeks: 75% vs. 67.8%	N/A	Endoscopy	20 mg vs. 30 mg	8 weeks	[60]
Vonoprazan vs. Lansoprazole (Laine 2023)	RE (grade C/D)	Healing at 2 weeks: 70.2% vs. 52.6%	N/A	Endoscopy	20 mg vs. 30 mg	2 weeks	[61]
Vonoprazan vs. Lansoprazole	EE	Faster heartburn relief and better nocturnal symptom control	Sleep quality improvement (PSQI)	Symptom diary and PSQI	20 mg vs. 30 mg	2 weeks	[59]

**Table 3:** Clinical outcomes of vonoprazan in NERD, GERD, and refractory RE [51,52,53,54,62].

Drug Name	Patient Group	Esophageal Response Rate	Extraesophageal Response Rate	Evaluation Method	Drug Dose	Treatment Duration	Reference Number
Vonoprazan	PPI-resistant RE	87.5% healing rate	N/A	Endoscopy and FSSG	20 mg	4 weeks	[51]
Vonoprazan	PPI-refractory RE	87.5% mucosal healing	N/A	Endoscopy, pH-metry, and HRM	20 mg	4 weeks (+additional 4 weeks if necessary)	[52]
Vonoprazan	GERD (erosive esophagitis + NERD)	89% symptom improvement at 1 month, 82% at 12 months	Improvement in throat discomfort, postprandial distress, diarrhea	Endoscopy and symptom scales	10 mg	12 months	[53]
Vonoprazan	GERD (erosive esophagitis + NERD)	86% symptom improvement, 57% complete remission at 1 month	N/A	Symptom evaluation	10 mg	1 month	[54]
Vonoprazan (on-demand)	NERD	56–70% complete symptom resolution vs. 27.3% placebo	N/A	Symptom questionnaires	10–40 mg (on demand)	6 weeks	[62]

**Table 4:** Meta-analyses and long-term outcomes with P-CAB therapy [63,64].

Drug Name	Patient Group	Esophageal Response Rate	Extraesophageal Response Rate	Evaluation Method	Drug Dose	Treatment Duration	Reference Number
Vonoprazan (meta-analysis)	RE (grade C/D)	SUCRA 0.89; treatment failure 6% vs. 21% (PPIs)	N/A	Meta-analysis of RCTs	20 mg	Various	[63]
Vonoprazan (VISION study)	RE (long-term)	Recurrence rate 10.8% vs. 38% with lansoprazole	N/A	Endoscopy (every 6 months)	20 mg	5 years	[64]

**Table 5:** Clinical outcomes of tegoprazan in erosive esophagitis, GERD, and NERD [65,66,67].

Drug Name	Patient Group	Esophageal Response Rate	Extraesophageal Response Rate	Evaluation Method	Drug Dose	Treatment Duration	Reference Number
Tegoprazan vs. Lansoprazole	EE	Healing at 4 weeks: 95.2% vs. 86.2%	N/A	Endoscopy	50 mg vs. 30 mg	4 weeks	[66]
Tegoprazan	NERD	42.5–48.5% complete symptom resolution vs. 24.2% placebo	N/A	Symptom scales	50 mg/100 mg	4 weeks	[67]
Tegoprazan	GERD (NERD + EE, on-demand)	26.2% rapid symptom relief vs. 16.1% with esomeprazole	N/A	Symptom scales	50 mg	8 weeks (on demand)	[65]

**Table 6:** Comparison of selected adverse events observed with vonoprazan and proton pump inhibitors (PPIs) based on integrated clinical and post-marketing data [69,70].

Adverse Effect	Frequency Range (%)	
	Vonoprazan	PPI
Nasopharyngitis	6.94%	5.07%
Diarrhea	2.69%	3.68%
Upper respiratory tract infection	1.96%	2.83%
Pancytopenia	1.73%	1.95%
Back pain	1.49%	0.94%
Gastroenteritis	1.43%	0.75%
Toxic epidermal necrolysis	1.42%	2.51%
Nausea	1.35%	0.99%
Constipation	1.20%	1.19%
Abdominal distension	1.17%	1.59%
Abdominal pain (upper)	1.13%	1.49%
Hypertension	1.13%	1.04%
Dyspepsia	1.09%	1.34%
Headache	1.00%	1.74%
Alanine transaminase increase	0.96%	1.49%
Dizziness	0.88%	1.14%

## Data Availability

No new data were created or analyzed in this study. Data sharing is not applicable to this article.

## Abbreviations

The following abbreviations are used in this manuscript:

ACG	American College of Gastroenterology
AEs	adverse events
CAP	community-acquired pneumonia
CI	confidence interval
DGER	duodeno-gastro-esophageal reflux
ECL	Enterochromaffin-like
EE	erosive esophagitis
FDA	Food and Drug Administration
FSSG	Frequency Scale for Symptoms of Gastroesophageal Reflux Disease
GERD	gastroesophageal reflux disease
H^+^/K^+^-ATPase	hydrogen–potassium ATPase
H2RAs	H2 receptor antagonists
HRM	high-resolution manometry
HRT	Holding Time Radio
JADER	Japanese Adverse Drug Event Report
LA	Los Angeles
LES	lower esophageal sphincter
MAD	multiple ascending dose
NERD	non-erosive reflux disease
NETs	neuroendocrine tumors
PCABs	potassium-competitive acid blockers
PPIs	proton pump inhibitors
PSQI	Pittsburgh Sleep Quality Index
RCT	randomized controlled trial
RE	reflux esophagitis
SAD	single ascending dose
SAEs	serious adverse events
SUCRA	Surface Under the Cumulative Ranking Curve
